# Dental Department of the University of Tennessee

**Published:** 1882-05

**Authors:** 


### DENTAL DEPARTMENT OF THE UNIVERSITY OF TENNESSEE.

The fourth annual commencement.

The number of matriculates for the session was thirty-two.

The degree of D.D.S. was conferred on the following members of the class:

| NAME. | RESIDENCE. | NAME. | RESIDENCE. |
|---|---|---|---|
| C. B. Johnson | Mississippi. | A. E. McGlothlin | Tennessee. |
| W. W. Grant | Tennessee. | J. R. George | Texas. |
| N. M Culbreth | North Carolina. | S. S. Shackleford | Texas. |
| John N. Griner | Tennessee. | P. D. Houston | Tennessee. |
| J. N. Greer | Georgia. | Chas. E. Hawley | Louisiana. |
| W. N. Leseur | Tennessee. | J. C. Grant | Tennessee. |
| John T. Ryan | Tennessee. | W. H. Cooke | Texas. |

